# Motivation Matters: Differing Effects of Pre-Goal and Post-Goal Emotions on Attention and Memory

**DOI:** 10.3389/fpsyg.2012.00404

**Published:** 2012-10-17

**Authors:** Robin L. Kaplan, Ilse Van Damme, Linda J. Levine

**Affiliations:** ^1^Department of Psychology and Social Behavior, University of CaliforniaIrvine, CA, USA; ^2^Laboratory of Experimental Psychology, Research Foundation – Flanders, University of LeuvenLeuven, Belgium

**Keywords:** emotion, arousal, memory narrowing, motivation, goal relevance

## Abstract

People often show enhanced memory for information that is central to emotional events and impaired memory for peripheral details. The intensity of arousal elicited by an emotional event is commonly held to be the mechanism underlying memory narrowing, with the implication that all sources of emotional arousal should have comparable effects. Discrete emotions differ in their effects on memory, however, with some emotions broadening rather than narrowing the range of information attended to and remembered. Thus, features of emotion other than arousal appear to play a critical role in memory narrowing. We review theory and research on emotional memory narrowing and argue that motivation matters. Recent evidence suggests that emotions experienced prior to goal attainment or loss lead to memory narrowing whereas emotions experienced after goal attainment or loss broaden the range of information encoded in memory. The motivational component of emotion is an important but understudied feature that can help to clarify the conditions under which emotions enhance and impair attention and memory.

Our most vivid and lasting memories are typically emotional ones. These memories are selective, however. People show excellent memory for information that is central to an emotional event, but poorer memory for peripheral details. Memory narrowing as a result of emotion has been demonstrated in numerous studies but sources of controversy remain. Researchers disagree about what constitutes central information versus peripheral detail, about whether emotion always leads to memory narrowing, and about underlying mechanisms. This review scrutinizes theory and research on memory narrowing and questions the common view that arousal is the primary mechanism underlying this effect. Inconsistencies in the literature are discussed, and alternative mechanisms are evaluated. We argue that several controversies can be clarified by the view that the goals or motivations associated with discrete emotions determine the breadth of cognitive processing. Taking into account the motivational function of emotions leads to a better understanding of when and why emotions enhance and impair cognition.

## Emotional Memory Narrowing

The terms “emotional memory narrowing” (Reisberg and Heuer, 2007), “tunnel memory” (Safer et al., 1998), and “central/peripheral trade-off” (Kensinger et al., 2007) all refer to the finding that people tend to remember information central to, or at the core of, an emotional event, and have poorer memory for peripheral or background features of the event (e.g., Christianson and Loftus, 1991). Emotional memory narrowing has been demonstrated most famously via the “weapon focus” effect. In laboratory studies in which a crime is staged, witnesses tend to focus on and remember the weapon used to commit the crime at the expense of memory for details such as the perpetrator’s face and clothing (e.g., Loftus et al., 1987; Kramer et al., 1990; Steblay, 1992). The weapon, or immediate source of danger, is the central and emotionally arousing stimulus whereas other information, such as the perpetrator’s face or clothing, is peripheral to the concern of remaining safe. Thus, emotion is associated with the narrowing of attentional resources to a central threat at the expense of peripheral or background details.

Considerable evidence for emotional memory narrowing has been obtained in other laboratory-based studies as well (for reviews, see Christianson, 1992; Mather, 2007; Kensinger, 2009; Levine and Edelstein, 2009). For example, Christianson and Loftus (1987) showed participants slides that depicted a traumatic event (a boy getting hit by a car) or a neutral event (a boy crossing a street) and tested memory for the slides later. Participants who saw the traumatic event displayed better memory for central features of the slides, but were worse at remembering which specific slides they had seen, relative to those who saw the neutral event. This suggests that people remember different aspects of events depending on whether or not the events evoked emotion (also, see Christianson, 1984; Christianson and Loftus, 1991). Similarly, Kensinger et al. (2007) had participants view emotion-eliciting objects (e.g., a snake) and neutral objects (e.g., a chipmunk) in front of neutral backgrounds (e.g., a river). Participants showed more detailed memory for emotional than neutral objects but remembered backgrounds paired with neutral objects better than backgrounds paired with emotional objects. Thus, emotion was associated with enhanced memory for central information at the expense of peripheral details.

Outside the laboratory, people have shown enhanced memory for central features of emotional events as varied as natural disasters (Bahrick et al., 1998), physical injuries (Peterson and Bell, 1996), crime scenes (Reisberg and Heuer, 2007), and personal events that evoked shock, anger, and fear (Berntsen, 2002). For instance, when asked to recount autobiographical events that were highly emotional, people showed enhanced memory for features that were integral to the events and essential to their meaning but poorer memory for peripheral details that made no difference to the meaning of the events (Talarico et al., 2009). In summary, a large body of research, both inside and outside the laboratory, indicates that emotion induces memory narrowing. Several unresolved issues remain, however. These include the aspects of emotion responsible for memory narrowing, whether all emotions lead to memory narrowing, and what constitutes central information versus peripheral detail. We turn to these issues next, reviewing approaches to emotional memory narrowing that emphasize arousal, valence, and goal relevance.

## Emotional Arousal and Memory Narrowing

Emotional arousal, a physiological state that ranges from calm relaxation to excitement and tension, is one of the major features of emotion (Russell, 2003), and is commonly considered to be the primary mechanism underlying memory narrowing. A high level of arousal results from activation of the autonomic nervous system and the release of stress hormones such as epinephrine and cortisol, and can be measured by indices such as heart rate and skin conductance. The view that emotional arousal induces narrowing of attention and memory dates back to the 1950s. Callaway and Dembo (1958) showed that people given stress hormones responded less to stimuli outside of their central line of vision. Similarly, Easterbrook’s (1959) cue-utilization hypothesis states that emotional arousal reduces the number of cues that an organism can process at a time, decreasing attention allocated to peripheral information. The greater the intensity of arousal, the narrower the attentional focus. Because of this narrow focus, encoding of central information is enhanced, whereas encoding of peripheral details is impaired (for reviews, see Reisberg and Heuer, 2007; Mather and Sutherland, 2011). This assists organisms in reaching current goals by blocking out information irrelevant to those goals (e.g., LaBar and Cabeza, 2006).

In addition to influencing attention and encoding, arousal has been shown to promote consolidation of emotional information in long term memory through activation of the amygdala (e.g., Cahill et al., 1994; van Stegeren et al., 1998; Nielson et al., 2005; Dolcos et al., 2011). Infusing norepinephrine directly into the basolateral amygdala after the experience of emotional events enhances long term memory for those events. Inactivating this region, using lesions or drugs, attenuates memory enhancement (McGaugh, 2004, 2006). Stressful circumstances leading to cortisol release prior to retrieval of an emotional event can also lead to more pronounced memory narrowing relative to less stressful conditions (Echterhoff and Wolf, 2012). Thus, arousal affects attention, encoding, consolidation, and retrieval, and is thought to underlie enhanced memory for central information and poorer memory for information that is peripheral to the emotional event.

## Findings Not Explained by the Arousal Model

Despite general agreement that arousal leads to memory narrowing, inconsistent results have been obtained. Emotion has been shown to enhance memory for both central and peripheral information (e.g., Heuer and Reisberg, 1990; Cahill and McGaugh, 1995; Libkuman et al., 1999; Dutton and Carroll, 2001; Laney et al., 2004), to impair memory for both central and peripheral information (e.g., Morgan et al., 2004), and to enhance memory for peripheral details (e.g., Fredrickson and Branigan, 2005; Talarico et al., 2009). Moreover, the effect of emotion on memory narrowing appears to be influenced by the valence of the event or stimulus (e.g., Berntsen, 2002; Waring and Kensinger, 2009; Mickley Steinmetz et al., 2010; van Steenbergen et al., 2011; Van Damme and Smets, submitted).

The most frequently reported violation of the arousal model of emotional memory narrowing is an enhancement of memory for both central and peripheral information. Laney et al. (2004) asked participants to view a series of slides that was rendered emotionally evocative or emotionally neutral via voice narration. Relative to participants in the neutral group, participants for whom the slides were placed in an emotionally arousing context reported greater subjective emotional intensity and showed greater physiological arousal. They also displayed enhanced memory for both central and peripheral features of the slides. Emotional arousal thus improved overall memory accuracy. These investigators argued that emotional memory narrowing may not occur without the presence of an “attention magnet” – a shocking and salient sensory stimulus such as a picture of a gruesome injury. They concluded that memory narrowing may not be an inevitable effect of emotional arousal but rather a byproduct of the type of shocking sensory stimuli most typically used to study the phenomenon.

A second set of findings conflicts with the view that emotional arousal enhances memory for central information by finding general memory impairment. For example, soldiers undergoing extremely stressful circumstances during military survival training (e.g., lack of food, sleep deprivation, and physical threats) were worse at identifying the faces of their interrogators than soldiers who underwent a less stressful interrogation (Morgan et al., 2004). A meta-analysis on the effects of stress on eyewitness memory also revealed that stress can impair memory for both the face of a perpetrator and details of the crime (Deffenbacher et al., 2004). In a spatial learning task, seeking monetary reward resulted in better memory than avoiding electrical stimulation, except when seeking reward led to high arousal (Murty et al., 2011). Thus, emotional arousal can lead to deficits in memory accuracy, including information of central importance to the emotion-eliciting event.

A third set of findings shows that emotional arousal can enhance, rather than impair, memory for peripheral details. For instance, Talarico et al. (2009) demonstrated that certain emotions (e.g., surprise, happiness, and sadness) were associated with greater recall of peripheral features of the emotion-eliciting events because they promoted broader reflection on the event as a whole. In addition, happiness and amusement have been found to enhance memory for global features of stimuli rather than leading to narrowing of attention or memory (e.g., Gasper and Clore, 2002; Fredrickson and Branigan, 2005).

Finally, the extent to which emotion leads to memory narrowing has been shown to vary depending on the valence of the emotion. Waring and Kensinger (2009) showed participants pictures of emotionally arousing objects placed within neutral backgrounds and pictures of neutral objects placed within neutral backgrounds. The valence (positive, negative) and the level of arousal (low, high) of the emotional objects were manipulated. Background memory was generally worse for emotionally arousing objects than for neutral objects, but the effect was weakest for positive low arousal objects. After a 24 h study-test delay, a central-peripheral memory trade-off remained only for negative high arousal pictures, not for positive high arousal pictures or for low arousal pictures of either valence (also, see Mickley Steinmetz et al., 2010; Yegiyan and Yonelinas, 2011). This suggests that valence and arousal interact in determining the extent of emotional memory narrowing. Berntsen (2002) obtained similar findings with respect to autobiographical memories, with accounts of shocking events, but not happy events, containing more central than peripheral information.

## Reasons for Inconsistent Findings

We have reviewed research showing that emotional arousal narrows the range of information attended to and encoded in memory as well as findings that contradict this view. To make sense of emotional memory narrowing, and of results that violate this pattern of findings, it is necessary to be more explicit about what constitutes high versus low levels of emotional arousal, and what constitutes information that is central as opposed to peripheral to an emotional event. It is also necessary to consider mechanisms other than arousal that may underlie accurate and lasting memory for central information and poor memory for peripheral detail.

### Varying intensities of emotion across studies

One reason for inconsistencies in the findings is consistent with the arousal model. The effect of emotion on memory may differ depending on the degree of arousal, but “high arousal” is defined very differently across studies. Many studies contrast memory for stimuli or events that are deemed emotionally arousing versus emotionally neutral. The stimuli and events defined as arousing, however, range from relatively mild laboratory stimuli (e.g., Laney et al., 2004) to real world events that evoke extreme levels of stress (e.g., Morgan et al., 2004). Thus, emotion may enhance memory generally when the level of arousal is relatively low, lead to memory narrowing when the level of arousal is moderate, and impair memory generally when the level of arousal is extremely high (Baldi and Bucherelli, 2005). To the extent that investigators neglect to contrast increasing levels of arousal, and define high arousal differently across studies, non-linear relationships between arousal and memory would be difficult to identify.

### Varying definitions of central and peripheral information

Another reason for inconsistencies in the findings concerning emotional memory narrowing may be that definitions of central and peripheral details vary widely across studies and theoretical frameworks (Kensinger, 2009; Levine and Edelstein, 2009). Central information has been variously defined as information that is spatially (e.g., Christianson and Loftus, 1991), temporally (e.g., Strange et al., 2003), or conceptually (e.g., Peterson and Whalen, 2001) integral to the emotion-eliciting event or stimulus, or as information that is relevant to the individual’s current goals (e.g., Levine and Edelstein, 2009). Information considered to be central in one study may be viewed as peripheral in another. For instance, in research on the weapon focus effect, the clothing of the perpetrator holding the weapon may be considered spatially integral, and hence central, due to its proximity to the weapon (e.g., Christianson and Loftus, 1991). Alternatively, clothing can be considered a peripheral detail because it is distinct from the weapon or central attention magnet (Laney et al., 2004) and has no significance for the goal of avoiding threat (e.g., Levine and Edelstein, 2009).

In an effort to more clearly define “central” versus “peripheral” information, Mather and Sutherland (2011, 2012) proposed the “arousal-biased competition” (ABC) model. According to this model, emotional arousal enhances encoding of high priority information at the expense of low priority information. Information can attain high priority as a result of bottom-up perceptual processes or as a result of top-down conceptual processes. For instance, recent work on the ABC model has demonstrated that when participants were exposed to a loud, arousing noise or to negatively arousing images, they were more likely to recall perceptually salient stimuli (high contrast symbols and letters) versus less salient stimuli (low contrast symbols and letters), compared to when they were exposed to a neutral noise or image (Lee et al., 2012; Mather and Sutherland, 2012). The perceptual contrast of the symbols and letters is an example of salience resulting from bottom-up perceptual processes. In addition, the ABC model posits that memory for information that is relevant to a person’s goals will be enhanced whereas memory for information that is not relevant will be impaired. This is an example of salience resulting from top-down processes. Thus, this model holds that emotional arousal enhances attention to, and memory for, information that has high priority at the expense of information that has low priority, regardless of whether the priority derives from bottom-up processes (perceptual salience) or top-down processes (goal relevance).

### Alternative mechanisms

As previously discussed, arousal is commonly viewed as the feature of emotion that leads to memory narrowing. However, a third reason for inconsistencies in the findings concerning emotional memory narrowing may be that features of emotion other than arousal play an important role in determining the range of information attended to and remembered. Investigations of alternative mechanisms are scarce, however, and potential mechanisms are often confounded in research on emotional memory narrowing. Turning again to studies of weapon focus, the sight of the weapon elicits physiological arousal, negative affect, the discrete emotion of fear, and the goal to protect the self from harm. Memory narrowing may be due to any one of these factors or to a combination of factors. Yet few studies experimentally manipulate one factor while holding other factors constant or controlling for them statistically (e.g., Ritchie et al., 2006). Thus, to account for findings that discrete emotions vary with respect to the range of information encoded in memory (e.g., Berntsen, 2002; Waring and Kensinger, 2009), it is necessary to consider other potentially important features of emotion. Two alternative approaches are discussed which suggest that features of emotion other than arousal influence the breadth of information attended to and remembered.

## Emotional Valence and Memory Narrowing

An alternative to the arousal model is the view that the valence of an emotion determines the type of information processing in which people engage and, as a result, the extent of memory narrowing. Kensinger (2009) reviewed evidence that negative emotion increases the processing and retrieval of sensory information, leading people to remember specific (central) details; in contrast, positive emotion increases the processing and retrieval of conceptual information, leading people to remember the gist of an event. In other words, negative emotion induces a focus on local details and therefore elicits a central-peripheral trade-off, whereas positive emotion leads to broadening of attention and reliance on gist or heuristics.

The affect-as-information model explains functions that may be served by these differing information processing strategies (e.g., Bless and Schwarz, 1999; Bless and Fiedler, 2006; Clore and Huntsinger, 2007; Clore and Palmer, 2009). According to this model, people use their affective state as a short-cut to infer their evaluative reactions to a situation, and this influences attention and memory. Negative emotion typically signifies that there is a problem to solve, and therefore signals the need to carefully monitor the environment for relevant information. Positive emotion signifies a safe situation in which no problems require immediate attention, so there is little need to focus on specific details. Hence, negative emotion triggers narrow item-specific or stimulus-driven processing, whereas positive emotion triggers broader relational or knowledge-driven processing. Extending this approach, Fredrickson’s (1998, 2001) broaden-and-build theory holds that positive emotions broaden people’s thought-action repertoires, promoting activities such as play, exploration, and integration of different types of information in the environment. This allows people to establish and build enduring cognitive, behavioral, and social resources. In contrast, negative emotions promote specific action tendencies (e.g., withdrawal when feeling fear) and narrow attention.

Empirical research on the attention broadening effects of positive emotion dates back to the 1980s. Isen et al. (1985, 1987) showed that positive emotion (induced through watching amusing film clips or receiving small gifts) enhances creativity and cognitive flexibility. More recently, positive emotion has been shown to enhance the accessibility of global information (Gasper, 2004), to promote attention to peripheral features of visual stimuli (e.g., Wadlinger and Isaacowitz, 2006; Rowe et al., 2007), and to broaden the scope of information encoded in memory (Gasper and Clore, 2002; Johnson and Fredrickson, 2005). For instance, Fredrickson and Branigan (2005) elicited positive, negative, or neutral affect in participants and then had them perform a global-local visual processing task (e.g., Navon, 1977; Kimchi and Palmer, 1982). Participants experiencing positive emotions (amusement or contentment) displayed a global attention bias relative to those experiencing neutral affect or negative emotions (anger or anxiety). This effect is thought to occur early on in visual processing (Kuhbandner et al., 2011).

Valence has also been shown to influence people’s susceptibility to memory distortion and false memories. By promoting heuristic processing, positive emotion can increase suggestibility and susceptibility to incorporating misinformation into memory; by promoting systematic processing, negative emotion can render people more resistant to misinformation (e.g., Bless et al., 1996; Levine and Bluck, 2004; Forgas et al., 2005; Storbeck and Clore, 2005; Kensinger and Schacter, 2006). In summary, although both positive and negative emotion can be accompanied by physiological arousal, some research indicates that negative emotion leads to narrowing of the range of information attended to and remembered, whereas positive emotion leads to broadening.

## Motivation or Goal Relevance and Memory Narrowing

Despite incorporating one of the most crucial differences among emotions, a focus on valence alone cannot account for some recent findings. Even emotions of the same valence and the same level of arousal have been shown to vary with respect to the range of information attended to and the degree of memory narrowing. Thus, a more complete understanding of the effects of emotion on memory may require moving beyond arousal and valence to consider the motivational state underlying discrete emotions (e.g., Sander et al., 2003; Larson and Steuer, 2009; Levine and Edelstein, 2009; Gable and Harmon-Jones, 2010b,c,d; Lench et al., 2011; Harmon-Jones et al., 2012a,b). The goal relevance model contains elements of both the arousal and valence approaches but makes more specific predictions about the types of emotions that should lead to memory narrowing. According to this model, the motivational intensity (Gable and Harmon-Jones, 2010d) or goal status (Levine and Pizarro, 2004; Levine and Edelstein, 2009) of an emotion determines the degree of memory narrowing.

### Motivation affects attention and memory

A wealth of research supports the view that people’s goals affect what they remember. Information relevant to a goal that has not yet been achieved remains more accessible in memory than the same information after the goal has been achieved (Förster et al., 2005). People remember information exceptionally well if it is framed in terms of enduring, universal goals such as survival (e.g., Nairne et al., 2008). In part, this occurs because people deliberately prioritize information relevant to active goals, putting more effort into encoding and retrieving it (e.g., Adcock et al., 2006). But memory enhancement for goal-relevant information also occurs automatically even when people are not instructed to remember the information or rewarded for doing so. In one study, participants showed enhanced performance on both an explicit memory task and an implicit memory task (facilitated access in lexical decision) for words that had been assigned a high point value. They also showed memory narrowing – poorer memory for the context in which high reward words had been learned. This occurred even though memory for words with high point values was not rewarded and no instructions to remember those words were given (Madan et al., 2012). Thus, consistent with prior research on the effects of emotional arousal on memory (e.g., Mather and Sutherland, 2011), these findings suggest that memory for goal-relevant stimuli is enhanced and that this benefit can occur at the expense of contextual details.

People’s goals differ depending on their developmental stage, personality traits, and coping styles. In each case, memory serves an adaptive function by retaining information that helps people reach their goals (Nairne et al., 2007). For example, older adults, who are motivated to regulate emotion to promote well-being, attend to and remember positive information more readily than younger adults (Mather and Carstensen, 2005). People high in extraversion and approach motivation (traits characterized by the goal to approach positive outcomes) remember reward-relevant stimuli better, and recall more positively valenced autobiographical memories, relative to people high in anxiety and introversion, who remember more threat-relevant information (e.g., Derryberry and Reed, 1994; Gable and Harmon-Jones, 2008; Denkova et al., 2012; Study 3; Hicks et al., 2012). With respect to coping, people recall self-enhancing information more readily than self-threatening information (Taylor and Brown, 1988; Sedikides and Green, 2009) and may selectively remember the past as worse than it actually was in order to bolster the belief that they have improved over time (e.g., Wilson and Ross, 2001; Ross and Wilson, 2003; Walker and Skowronski, 2009). People with an avoidant attachment style, characterized by the goal of avoiding intimate relationships with others, display impaired memory for relationship-relevant words on a working memory task (Edelstein, 2006).

This research shows that people’s goals, whether universal or individual, systematically shape what they attend to and remember. Motivation is a critically important component of emotion and may account to the effects of emotion on cognitive processing. Goals associated with discrete emotions (e.g., the goal of avoiding threat when feeling fear) may promote processing of goal-relevant information and impair processing of information that is peripheral to the goal. Thus, motivation or goal relevance represents an alternative mechanism that may account for emotional memory narrowing.

### The contribution of motivation to emotional memory narrowing

According to appraisal theories, goals are at the heart of what it means for an event to be emotional. People continuously monitor the environment for information relevant to their current goals or well-being (e.g., Scherer, 1998). They experience emotions when they perceive a change in the status of a goal that makes it necessary for them to modify their beliefs or plans. This motivational component of emotion may contribute to memory narrowing. Levine and Edelstein (2009) distinguish between pre-goal and post-goal emotions. Pre-goal emotions such as desire, excitement, fear, and anger, reflect appraisals that goal attainment or failure may occur in the future but have not yet occurred or that goal-directed efforts are ongoing. Post-goal emotions, such as happiness, contentment, sadness, and grief, reflect appraisals that goal attainment or failure has already occurred. Gable and Harmon-Jones (2010d) make a similar distinction when they refer to “motivational intensity.” High motivational intensity is experienced when a person is driven to approach or avoid specific stimuli in the environment because goal attainment or failure is anticipated but has not yet occurred.

Because information processing capacity is limited, the information most relevant to a person’s goals in a given context is likely to be noticed and remembered, whereas less relevant information is likely to be ignored or quickly forgotten. When motivational intensity is high, it is functional to attend to and remember information that is relevant to the active goal at the expense of less relevant information. However, when goal attainment or failure has already occurred, it is functional to attend to and remember a broader range of information. This allows the person to take into account the consequences of success or failure, change their beliefs and expectations, and orient toward possible new goals. In other words, emotions reflect the status of goals, and influence the scope of attention and the range of information encoded in memory accordingly. If goal attainment or failure is anticipated, the range of stimuli attended to and encoded in memory narrows to central, goal-relevant information. After goal attainment or failure, the range of stimuli attended to and encoded in memory broadens to incorporate more peripheral information. Importantly, according to this model, even if pre-goal and post-goal emotions elicit similar levels of arousal, they should differ in their effects on attention and memory. Moreover, valence only exerts an influence through goal relevance or motivational intensity: People typically feel negative emotion when goals are threatened and positive emotion when goals have been achieved. Nevertheless, both pre-goal and post-goal emotions can be either positive or negative.

Although studies investigating the effects of emotional valence on subsequent cognition typically assess positive emotion following goal attainment (e.g., receiving a gift, watching amusing film clips, recalling past successes), people also feel a range of positive emotions when they *anticipate* attaining goals (e.g., desire, enthusiasm, excitement). A number of researchers, using different terminologies, have described distinctions between pre-goal and post-goal positive affective states, and have demonstrated that they have different effects on subsequent cognition (Panksepp, 1998; Knutson et al., 2000; Berridge, 2006; Knutson and Wimmer, 2007; Levine and Edelstein, 2009). Berridge and colleagues distinguish between “wanting” and “liking.” Wanting is associated with left frontal and nucleus accumbens activation, along with the release of dopamine, which enhances the desire to seek reward (e.g., Adcock et al., 2006; Knutson and Wimmer, 2007; Harmon-Jones et al., 2008). Liking is associated with opioid stimulation, which is related to feelings of pleasure following goal attainment (Berridge and Robinson, 2003; Berridge and Kringelbach, 2008). Similarly, Panksepp (1998) distinguishes between the seeking versus playing emotive systems. Seeking aids goal-pursuit by narrowing attention to goal-relevant information (e.g., locating food or a mate). Playing promotes exploration of new territory and affiliation with others. Thus, pre-goal and post-goal positive emotions differ with respect to neural activity, the functions they serve, and breadth of processing.

Empirical studies support the view that pre-goal positive emotions promote memory narrowing. Gable and Harmon-Jones (2008) induced participants to feel either pre-goal desire or post-goal happiness. This was done by having participants view a film clip of delicious desserts or a humorous film clip. They found that desire narrowed attentional focus relative to happiness in global-local visual processing tasks. Other researchers found similar results when the desire to approach objects was elicited by displaying erotic stimuli (Moyer, 2004, unpublished study cited in Reisberg and Heuer, 2004), or by displaying stimuli that evoke feelings of nurturance, such as faces of human infants (Brosch et al., 2008), and animal infants (Gable and Harmon-Jones, 2008). Gable and Harmon-Jones (2011) also manipulated pre- and post-goal positive emotion within the same participants, and concerning the same goal, by giving participants the opportunity to win money in a game. Positive emotion experienced in anticipation of winning led to narrowing of attention, whereas positive emotion experienced after winning led to broadening of attention. Importantly, this effect extends to memory. Relative to people experiencing post-goal positive emotion, people experiencing pre-goal positive emotion remembered words presented in the center of a screen better than those presented peripherally (Gable and Harmon-Jones, 2010b; for a review, see Harmon-Jones et al., 2012a).

Just as not all positive emotions are associated with broadening of attention and memory, not all negative emotions are associated with narrowing of attention and memory. For instance, von Hecker and Meiser (2005) found that people in a depressed mood paid attention to, and later remembered, more irrelevant as opposed to central information on a source monitoring task in comparison to people who were not feeling depressed. Similarly, Gable and Harmon-Jones (2010a) showed that sadness, an emotion evoked by appraising goal failure as irrevocable, broadened attention in global-local figures tasks. Sad participants responded faster to global features of the figures than to details relative to those in a neutral mood. Thus, evidence suggests that post-goal emotion, whether positive or negative, broadens the range of stimuli attended to and encoded in memory.

In contrast, pre-goal negative emotions, such as disgust, fear, and anger, narrow attention, allowing people to concentrate on current goals such as avoiding or eliminating potential threat (Moons and Mackie, 2007; Levine and Edelstein, 2009; Gable and Harmon-Jones, 2010a; Finucane, 2011). This closely mirrors the effects of pre-goal positive emotions, activating the same brain regions and narrowing the range of stimuli attended to and encoded in memory (Berridge, 2006). Amygdala activation, previously assumed to be associated primarily with detection of threatening stimuli, has also been linked with the elicitation of pre-goal positive emotions such as desire and with the detection of rewarding stimuli. This suggests that the function of the amygdala can be viewed more broadly as preparing people to approach or avoid specific goals or outcomes (Cunningham and Brosch, 2012).

## Summary

Approaches to understanding the effects of emotion on attention and memory differ substantially in their predictions. The predominant view in the literature is that when experiencing emotional arousal, the range of stimuli attended to and encoded in memory narrows toward central information. Hence, any source of emotional arousal should result in attention and memory narrowing. Not all findings in the literature support this view, however. Conflicting findings have led researchers to investigate mechanisms other than arousal that may explain when and why emotion enhances versus impairs cognitive processes. The view that emotional valence determines the breadth of cognitive processing explains some but not all recent findings. Even emotions of the same valence and the same level of arousal have been shown to vary with respect to the range of information attended to and remembered (e.g., Gable and Harmon-Jones, 2010b). Taking into account the motivational component of emotion helps to explain inconsistent findings in the literature.

Recent evidence suggests that the effect of emotion on memory differs depending on the goal status associated with the emotion (e.g., Levine and Edelstein, 2009; Gable and Harmon-Jones, 2010d). When emotional arousal is elicited by the goal of pursuing or avoiding a particular outcome, the range of details attended to and encoded in memory narrows, with a focus on central or goal-relevant information. In contrast, when emotional arousal is experienced as a result of goal attainment or loss, the range of details attended to and encoded in memory broadens, resulting in enhanced memory for more peripheral features of events or stimuli. This broader perspective facilitates building on goal attainment or adjusting to goal failure. In sum, pre-goal emotions promote a narrow focus on information that will facilitate goal attainment or prevent loss, shutting out irrelevant details. Post-goal emotions help people adjust to goal attainment or loss, and orient toward possible new goals, by broadening the range of information attended to and remembered.

## Future Directions

Previous research has focused largely on how arousal and valence influence attention and memory. We have argued that investigating features of emotion beyond arousal and valence yields a more thorough understanding of how emotions influence attention and memory (Levine and Pizarro, 2004; Larson and Steuer, 2009; Levine and Edelstein, 2009; Gable and Harmon-Jones, 2010a; Lench et al., 2011; Harmon-Jones et al., 2012b). In the future, researchers should systematically vary the goal status of emotion as well as examining the effects of arousal and valence on attention and memory narrowing (see Ritchie et al., 2006; Larson and Steuer, 2009). In addition, varying the relevance of the information to be remembered will help to determine whether information relevant to current goals is enhanced at the expense of irrelevant information.

Research is also needed on the extent to which the types of information considered to be of central importance differ across discrete emotions. Central information to a person feeling desire might consist of rewarding and appetitive stimuli (e.g., Gable and Harmon-Jones, 2008). Central information to someone experiencing anger may be the perpetrator who is obstructing the person’s goals (Levine and Pizarro, 2004). Threat-related information may be central to someone experiencing fear (e.g., Lench and Levine, 2005). Drawing on a motivational approach, one would also expect pre- and post-goal emotional states to differentially influence people’s susceptibility to false memories. Pre-goal emotions should promote more careful and focused information processing than post-goal emotions, decreasing the likelihood of memory distortion for central or goal-relevant information. This has yet to be examined and provides an interesting avenue for further study. Finally, research examining neural correlates of emotional memory has begun to shed light on the underlying mechanisms involved in encoding and retrieving emotional information (e.g., Adcock et al., 2006; Dolcos et al., 2011, 2012; Shafer et al., 2011), and constitutes an important avenue for further research distinguishing the effects of pre- versus post-goal emotions on attention and memory. These are promising approaches that will help pinpoint how the motivational component of emotion shapes memory, and more broadly, the conditions under which emotion enhances versus impairs cognition.

## Conclusion

The motivational component of emotion is an important but understudied issue in the emotional memory narrowing literature. Future research on how motivation and goal relevance influence attention and memory will help clarify why, and under what conditions, people attend to and recall different types of information. Understanding the interaction between emotion and memory is a fundamental issue in the field of psychology. People are faced daily with the need to remember information while experiencing a range of emotional states. An attorney angered by the acts of opposing counsel, a patient saddened by a diagnosis, a rescue worker frightened by a disaster, must encode and retrieve detailed information accurately if they are to make good decisions. The link between people’s emotions and their goals provides an important key to understanding the selective nature of memory for emotional events. Arousal is an essential component of emotion which certainly affects attention and memory. But people frequently experience specific emotions such as happiness, desire, grief, and fear at high levels of arousal, yet they differ with respect to the types of information they are likely to attend to and the scope of their memories. Thus, taking into account the goal status and information processing strategies associated with discrete emotions can provide a more complete understanding of how emotion enhances and impairs cognitive processes.

## Conflict of Interest Statement

The authors declare that the research was conducted in the absence of any commercial or financial relationships that could be construed as a potential conflict of interest.
